# Targeting CD47 and Angiogenesis Demonstrates Effective Anti-Tumor Effect in Bladder Cancer

**DOI:** 10.3390/biomedicines12092152

**Published:** 2024-09-23

**Authors:** Xiting Huang, Qian Wang, Yanyang Nan, Xuyao Zhang, Ke Xu, Dianwen Ju, Weihong Ding

**Affiliations:** 1 Department of Biological Medicines and Shanghai Engineering Research Center of Immunotherapeutics, School of Pharmacy, Fudan University, Shanghai 201203, China; xthuang22@m.fudan.edu.cn (X.H.); wangqian23@m.fudan.edu.cn (Q.W.); yynan20@fudan.edu.cn (Y.N.); xuyaozhang@fudan.edu.cn (X.Z.); 2 Department of Urology, Huashan Hospital, Fudan University, 12 Central Urumqi Road, Shanghai 200040, China; drkexu@163.com

**Keywords:** bladder cancer, CD47 blockade therapy, innate immunity, angiogenesis, combinational therapy

## Abstract

**Background:** Although immunotherapy has shown potential in cancer treatment, current immunotherapeutics for bladder cancer are limited by a low response rate. Therefore, it is necessary to investigate other suitable immunotherapeutic targets and strategies for bladder cancer. **Methods**: To evaluate whether CD47 could be a suitable target for bladder cancer immunotherapy, CD47 protein expression levels in 116 bladder cancer tissue samples were assessed by IHC staining. In vitro anti-tumor effect of blocking CD47 was examined by phagocytosis assays. In vivo anti-tumor effects of targeting CD47 and angiogenesis were experimented in the HSPCs-CDX model. **Results**: We find that CD47 is highly expressed in bladder cancer samples and is associated with poor prognosis. Blocking CD47 could enhance the human PBMC-derived macrophages’ phagocytosis of T24 (from 10.40% to 29.70%) and 5637 (from 5.31% to 33.52%) human bladder cancer cells, as well as demonstrate anti-tumor effects in the HSPCs-CDX model (tumor growth inhibition rate, TGI: 33.05%). During CD47 treatment, we observed that the level of angiogenesis increased after CD47 blockade, and it might undermine the effect of CD47 immunotherapy. We then combined CD47 blockade with anti-angiogenic drugs to treat bladder cancer and discovered that inhibiting angiogenesis could further improve the anti-tumor effect of CD47 blockade (TGI: 76.39%). Finally, we tested the anti-tumor effect of co-targeting CD47 and angiogenesis using a bispecific fusion protein, SIRPα-VEGFR1, which successfully inhibited tumor growth to a similar extent as a combination therapy. **Conclusions:** Our study suggests that targeting CD47 could inhibit the growth of bladder cancer by promoting macrophage-mediated anti-tumor immunity. Moreover, blocking CD47 and angiogenesis could achieve a potent anti-tumor effect and could be an effective immunotherapy strategy for bladder cancer.

## 1. Introduction

Bladder cancer is a common urinary tract neoplasm and a prevalent cancer worldwide [1]. However, few advances have been achieved in bladder cancer treatment over the past three decades. Cisplatin-based neoadjuvant/adjuvant chemotherapy remains the most recommended regimen for controlling progression and recurrence, yet less than 50% of patients tolerate and respond to this treatment [2,3]. To overcome this therapeutic limitation, researchers and clinicians have set their sights on immunotherapy. The success of Bacillus Calmette–Guerin (BCG) intravesical instillation for early-stage bladder cancer demonstrated the feasibility of immunotherapy in bladder cancer and supported the usage of systemic immunotherapy in advanced-stage bladder cancer. Results from clinical trials also showed that patients could benefit from systemic immunotherapy [4]. Currently, systemic immunotherapies have been approved as alternative agents for cisplatin-intolerant patients to regain response and improve survival rates [5], but the objective response rate is still just around 20% [6]. Therefore, novel treatment targets and strategies are needed to achieve better responses in the immunotherapy of bladder cancer.

CD47 is a phagocytosis checkpoint which is broadly expressed in the body [7] via interaction with signal regulatory protein alpha (SIRPα) expressed on macrophages and other myeloid cells; it transmits “don’t eat me” signals to prevent healthy cells from immune clearance [8,9]. However, overexpression of CD47 has been discovered in numerous solid tumors and hematologic malignancies, for example, breast cancer, non-small cell lung cancer (NSCLC), and lymphoma, which can lead to immune escape and forecasts poor outcomes [10,11]. This makes CD47 a promising target for tumor immunotherapy. In many preclinical studies, blocking the CD47-SIRPα axis has been proven to enhance the macrophage-mediated killing of tumor cells, resulting in tumor growth inhibition [12,13,14,15,16,17]. Despite the achievement of anti-CD47 therapy in different cancers, few studies have been conducted to assess the remedial potential of targeting CD47 in bladder cancer. It remains unclear whether CD47 is a suitable target for bladder cancer.

Immunotherapy alone has displayed decent and passable efficacy. However, various factors, for example, the complex immunosuppressive tumor microenvironment and dose-dependent inflammatory toxicities, could hinder the application and success of immunotherapy [18,19]. Angiogenesis is a universal phenomenon across solid tumors which results from the elevated levels of certain pro-angiogenic factors like VEGFA [20,21]. By generating abnormal vessels, the dysfunctional vascular system supports the growth of tumors and creates an immunosuppressive tumor microenvironment [22,23]. In a wide range of cancers, angiogenesis has been found to be one of the major hurdles to responsiveness in PD-1/PD-L1 blockade therapy, limiting the infiltration and function of anti-tumor immune cells and resulting in a suboptimal anti-tumor effect [24,25,26]. The combination of anti-angiogenic drugs with PD-1/PD-L1 blockades has demonstrated the ability to overcome the immunosuppressive impact and carry out an optimal synergistic anti-tumor effect [22,24]. As a result, the FDA has approved a number of combination regimens for the treatment of NSCLC and hepatocellular carcinoma (HCC), which are proven to be successful in prolonging patients’ survival [22,27]. Further studies are worth carrying out to extend the application of this promising combination theory.

In this study, we aimed to evaluate the potential of targeting CD47 and angiogenesis in bladder cancer. Through analyzing 116 bladder cancer tissue samples, we studied CD47 protein expression levels in bladder cancer. We then conducted relevant in vitro and in vivo experiments to examine the anti-tumor effect of CD47 blockade in bladder cancer. We also investigated the role of angiogenesis in CD47 blockade therapy, and we further explored whether combining CD47 blockade with anti-angiogenic drugs could deliver a stronger anti-tumor effect in bladder cancer.

## 2. Materials and Methods

### 2.1. Cell Lines and Cell Culture

T24, a high-grade invasive human bladder cancer cell line, and 5637, a low-grade human bladder cancer cell line, were purchased from the National Collection of Authenticated Cell Cultures (Shanghai, China). Both cells were cultivated at 37 °C in a 5% CO_2_ incubator and cultured in RPMI 1640 medium (Meilunbio, # MA0215, Dalian, China) with 10% FBS (Gibco, #10099141C, Grand Island, MD, USA) and 1% Pen-Strep (Servicebio, # G4003-100ML, Wuhan, China).

### 2.2. Fusion Proteins

Three fusion proteins were kindly gifted from ImmuneOnco Biopharmaceuticals (Shanghai) Co., Ltd. (Shanghai, China). They were (a) SIRPα-Fc, a recombinant human signal regulatory protein α IgG1 fusion protein; (b) VEGFR1-Fc, a recombinant human vascular endothelial growth factor receptor 1 IgG1 fusion protein; (c) SIRPα-VEGFR1, a fusion protein which consists of SIRPα, and VEGFR1 (GenBank accession number: MG920788).

### 2.3. Phagocytosis Assays

Peripheral blood mononuclear cells (PBMCs) were separated from healthy donor peripheral blood using human lymphocyte separation medium (Dakewe, #7111011, Shenzhen, China) and were incubated with GM-CSF (50 ng/mL) (Abclonal, #RP00094, Wuhan, China) for 7 days to obtain macrophages. A total of 5 × 10^4^ of T24 or 5637 bladder cancer cells labeled with CFDA SE (Beyotime, #C0051, Shanghai, China) were preincubated with human IgG1 isotype control (5.3 μg/mL) (Selleck, #A2051, Houston, TX, USA) or SIRPα-Fc (1, 5, and 10 μg/mL) in 96-well plates for 30 min. A total of 5 × 10^4^ human macrophages were then added to the plate and cocultured with the tumor cells for 2 h. After co-incubation, whole cells were stained with PE anti-CD11b (BioLegend, #101208, San Diego, CA, USA) or PerCP anti-CD11b (BioLegend, #101229, San Diego, CA, USA); stained samples were then analyzed via CytoFLEX S Flow Cytometer (Beckman, Indianapolis, IN, USA). The phagocytosis was assessed by evaluating the ratio of CD11b^+^ CFSE^+^ phagocytic macrophages.

### 2.4. Flow Cytometry

For surface staining, cells were collected and blocked using 5% BSA for 30 min at 4 °C. Then, the cells were washed with PBS, followed by incubation with the corresponding florescence-labeled antibodies for 30 min at 4 °C. After staining, cells were washed at least twice with PBS before analyzed via CytoFLEX S Flow Cytometer (Beckman, IN, USA). The data were analyzed using CytExpert 2.4. The gating strategy for phagocytosis assays can be found in Figure S1.

### 2.5. Cell Line-Derived Tumor Xenograft (CDX) Model in Hematopoietic Stem and Progenitor Cells (HSPCs)-Derived Humanized Mice

Six-week-old male NCG mice were purchased from Shanghai Model Organisms Center, Inc. and housed under specific pathogen-free conditions. NCG mice were used to generate the HSPCs-CDX model. NCG mice were intraperitoneally injected with Busulfan (25 mg/kg) 24h before human CD34^+^ hematopoietic stem cell transplantation. CD34^+^ cells were isolated from neonate cord blood by a CD34-positive selection kit (STEMCELL Technologies, #17897, Seattle, WA, USA) and resuspended in PBS at a concentration of 2 × 10^5^ cells/mL. Each mouse was intravenously transplanted with 2 × 10^4^ of CD34^+^ cells (100 μL suspension). Two weeks after transplantation, peripheral blood was collected and stained with PerCP anti-human CD45 (BioLegend, #304025, CA, USA); human CD45^+^ cells could be detected by flow cytometry. T24 cells were collected and resuspended in PBS at a concentration of 1 × 10^8^ cells/mL. A total of 1 × 10^7^ T24 cells (100 μL suspension) were subcutaneously injected into the flank of each mouse (T24 cells were chosen for in vivo experiments due to the stability of tumorigenicity).

To investigate the anti-tumor effect of CD47 blockade in bladder cancer, mice were randomly divided into two groups (n = 5/group) when tumor average volume reached 100 mm^3^ and were dosed as follows: 2.4 mg/kg human IgG1 isotype control and 4.5 mg/kg SIRPα-Fc were injected intraperitoneally twice a week for 28 days. Xenografted tumor samples were isolated on day 28.

To investigate the anti-tumor effect of combining anti-angiogenic drugs with CD47 blockade in bladder cancer, mice were randomly divided into four groups (n = 5/group) when tumor average volume reached 100 mm^3^ and were dosed as follows: 2.4 mg/kg human IgG1 isotype control, 4.5 mg/kg SIRPα-Fc, 3.5 mg/kg VEGFR1-Fc, and 4.5 mg/kg SIRPα-Fc plus 3.5 mg/kg VEGFR1-Fc were injected intraperitoneally twice a week for 28 days. Xenografted tumors samples were isolated on day 28.

To investigate the anti-tumor effect of co-targeting CD47 and angiogenesis in bladder cancer, mice were randomly divided into three groups (n = 5/group) when tumor average volume reached 100 mm^3^ and were dosed as follows: 2.4 mg/kg human IgG1 isotype control, 4.5 mg/kg SIRPα-Fc plus 3.5 mg/kg VEGFR1-Fc, and 5 mg/kg SIRPα-VEGFR1 were injected intraperitoneally twice a week for 28 days. Xenografted tumor samples were isolated on day 28.

Tumor volumes were recorded twice a week and calculated as follows: tumor width^2^ × tumor length/2. Mice were euthanized if the tumor volume reached 2000 mm^3^ or exhibited body weight loss greater than 20% of the initial body weight. After anesthetizing mice with isoflurane, euthanasia was performed by cervical dislocation.

### 2.6. Immunohistochemistry and Immunofluorescence Staining

Paraffin-embedded patient tissue samples were provided by the Department of Urology, Huashan Hospital, Fudan University. Control bladder tissues were obtained from patients with cystitis. Pathological examinations of the samples have been performed by the pathologists. Characteristics for 116 patients with bladder cancer can be found in Table S1. Tumor samples from in vivo experiments were fixed in 4% paraformaldehyde, dehydrated by ethanol gradient, then embedded in paraffin. Paraffin-embedded samples were generated and processed into 4 μm tissue sections. Deparaffinization, antigen retrieval, and blocking were performed before staining. The sections were first incubated with primary antibodies (an anti-CD47 antibody [Abcam, #ab218810, Cambridge, MA, USA], a VEGF-A monoclonal antibody [Proteintech, #19003-1-AP, Wuhan, China], an anti-CD31 antibody [Servicebio, #GB113151, Wuhan, China], and an anti-CD68 antibody [Servicebio, #GB113150, Wuhan, China]) overnight at 4 °C. For immunohistochemistry (IHC) staining, the sections were incubated with HRP polymer-conjugated secondary antibodies the next day and then developed using DAB chromogen. The sections were imaged using Olympus VS200 Slide Scanner (Olympus, Tokyo, Japan) and the IHC scores were automatically quantified in the regions of interest using the IHC profiler plugin in ImageJ software (version 1.53u) [28]. For immunofluorescence (IF) staining, the sections were incubated with the corresponding secondary antibodies. After that, the sections were counterstained with DAPI, then treated with a spontaneous fluorescence quencher and mounting medium. The sections were imaged using Olympus SpinSR10 (Olympus, Tokyo, Japan). Applications of antibodies can be found in Table S2.

### 2.7. Intratumoral Cytokine Detection

Tumor tissues were homogenized using 3.2 mm grinding beads and Tissuelyser-24L (Jingxin, Shanghai, China) in cold RIPA. Tumor lysate supernatant was collected after centrifugation at 14,000× *g* for 10 min at 4 °C. The levels of IL-10 and TNF-α were measured by ELISA kits (Human TNF-α ELISA Kit [Multi Sciences, # EK182-96, Hangzhou, China], Human IL-10 ELISA Kit [Multi Sciences, # EK110/2-96, Hangzhou, China]) according to the manufacturer’s instructions.

### 2.8. Analysis of Public Datasets

Data from patients and animals were obtained from previously reported sources and references in publications. Gene expression data of IMvigor210 study were obtained from http://research-pub.gene.com/IMvigor210CoreBiologies/ (accessed on 29 September 2023). Data were extracted and normalized to TPM values and integrated with detailed clinical annotations using R studio (version 4.2.2). A comparison of CD47 expression between different T stages and immune phenotypes of bladder cancer were analyzed by nonparametric one-way ANOVA using GraphPad Prism 8. Nanostring data for PBS-treated or anti-CD47-treated MC38 tumor samples (GSE177053) were downloaded from the GEO database. Data were normalized according to Nanostring analysis guidelines (https://nanostring.com/wp-content/uploads/Gene_Expression_Data_Analysis_Guidelines.pdf; accessed on 7 October 2023). Differential expression of PECAM1 and VEGFA were analyzed by the Mann–Whitney test using GraphPad Prism 8.

### 2.9. Statistical Analysis

Statistical analyses were conducted in GraphPad Prism 8. Data normality was analyzed by the Gaussian distribution test; the equality of variance was analyzed by the F test and Brown–Forsythe test. A two-tailed unpaired Student’s t-test, the Mann–Whitney test, or one-way ANOVA was used to determine the significance for groups comparisons. The correlation between two variables was assessed by Spearman’s correlation analysis. *p*-values < 0.05 were considered to be statistically significant and were presented by asterisks as follows: * *p* < 0.05 and ** *p* < 0.01.

## 3. Results

### 3.1. CD47 Is Upregulated in Bladder Cancer and Is Associated with Poor Prognosis

**Figure 1:**
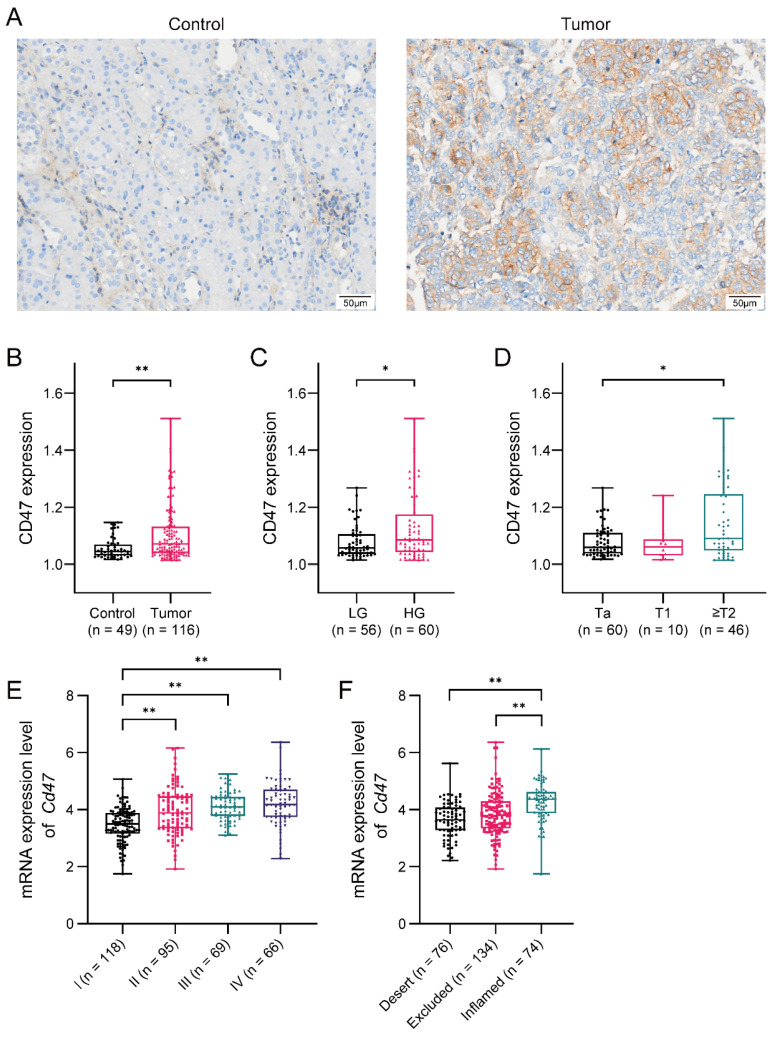
CD47 is a potential target for bladder cancer immunotherapy. (**A**) Representative images of CD47 immunohistochemistry staining of control and malignant human bladder tissues (magnification: ×10, scale bar: 50 μm). (**B**) Expression of CD47 in bladder cancer tissues and control bladder tissues. (**C**) Expression of CD47 between high-grade and low-grade bladder cancer. (**D**) Expression of CD47 between different T stages of bladder cancer. Screening CD47 mRNA expression in different T stages (**E**) and immune phenotypes (**F**) of bladder cancer from the IMvigor210 cohort. Data above were presented as boxplots and analyzed by the Mann–Whitney test and nonparametric one-way ANOVA. *p*-values < 0.05 were considered to be statistically significant and are presented by asterisks as follows: * *p* < 0.05 and ** *p* < 0.01.

We first analyzed the expression level of CD47 in bladder cancer tissues from 116 patients using IHC staining. Compared to control bladder tissues from patients with cystitis, CD47 expression is higher in bladder tumor tissues (Figure 1A,B). We further investigated CD47 expression levels in different tumor stages and grades and observed that CD47 is highly expressed in high-grade bladder cancer and muscle-invasive bladder cancer (Figure 1C,D), which suggests that CD47 may be clinically associated with poor prognosis in bladder cancer. To validate the observations from IHC staining, we screened the mRNA expression of CD47 in a bladder cancer cohort, IMvigor210. Results showed that CD47 mRNA expression was positively associated with a poor pathologic stage (Figure 1E). We also witnessed that CD47 mRNA expression is higher in the “inflamed” tumor, which implies that CD47 might act as a negative feedback factor to counter anti-tumor immunity (Figure 1F). Altogether, CD47 was upregulated and associated with poor prognosis in bladder cancer, indicating that it is a potential immunotherapy target for bladder cancer.

### 3.2. Targeting CD47 Demonstrates Anti-Tumor Effects in Bladder Cancer

**Figure 2:**
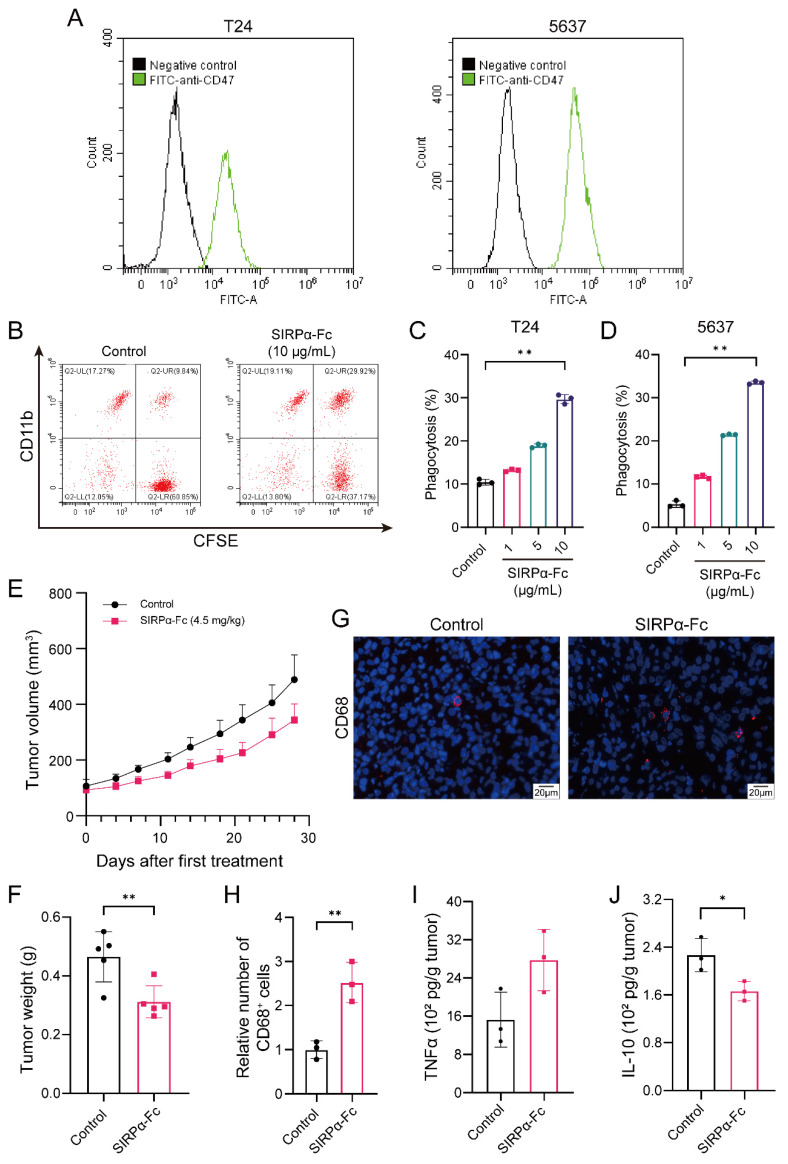
Blocking CD47 demonstrates macrophage-mediated anti-tumor activity in a bladder cancer CDX model. (**A**) Expression levels of CD47 on two human bladder cancer cell lines were detected by flow cytometry after staining with FITC anti-human CD47 (BioLegend, #323106, CA, USA). (**B**) Representative flow cytometry results exhibiting the phagocytosis of CFSE-labeled bladder cancer cells by human PBMC-derived macrophages in the incubation of the control antibody or SIRPα-Fc. Quantification of phagocytosis of T24 (**C**) and 5637 (**D**) bladder cancer cells in the incubation of human IgG1 isotype control (5.3 μg/mL) or SIRPα-Fc (1, 5, and 10 μg/mL) for 2.5 h. (**E**) Tumor growth rate was measured in the 4-week treatment with either human IgG1 isotype control (2.4 mg/kg, i.p. injection twice a week) or SIRPα-Fc (4.5 mg/kg, i.p. injection twice a week) in the HSPCs-CDX model. (**F**) Mice were euthanized on day 28 and tumor weight was recorded. (**G**,**H**) Representative IF images and the relative number of tumor-infiltrating macrophages from the HSPCs-CDX model after treatment with either human IgG1 isotype control or SIRPα-Fc (magnification: ×20, scale bar: 20 μm). (**I**,**J**) Intratumoral levels of TNF-α and IL-10 after treatment with either human IgG1 isotype control or SIRPα-Fc. Data above were presented as the mean ± SD and analyzed by two-tailed unpaired Student’s *t*-test. *p*-values < 0.05 were considered to be statistically significant and are presented as asterisks as follows: * *p* < 0.05 and ** *p* < 0.01.

After determining that CD47 was a potential target for bladder cancer, we started to investigate the anti-tumor activity of blocking CD47 in bladder cancer. It is known that CD47 helps tumor cells escape from the engulfment of macrophages [8], while interfering CD47-SIRPα interaction could restore the macrophage-mediated phagocytosis of tumor cells. Thus, we performed in vitro phagocytosis assays to assess whether the phagocytosis of CD47-expressing bladder cancer cells by human macrophages could be restored after CD47 blockade with the SIRPα-Fc fusion protein. Compared with the control antibody treatment, SIRPα-Fc treatment led to significant macrophage phagocytosis of bladder cancer cells expressing CD47 (*p* < 0.01) (Figure 2A–D), confirming the in vitro anti-tumor activity of blocking CD47. We then evaluated the in vivo therapeutic efficacy of SIRPα-Fc in the HSPCs-CDX model, which could better simulate the anti-tumor effect of CD47 blockade in a human immune system. Delayed tumor growth was observed in mice treated with SIRPα-Fc twice a week (Figure 2E,F). We further analyzed the changes in the tumor infiltration of immune cells and the intratumoral levels of cytokines. SIRPα-Fc administration resulted in increased amounts of tumor-infiltrating macrophages (Figure 2G,H), enhanced TNF-α production (Figure 2I), and a reduction in IL-10 expression (Figure 2J). These results suggest that targeting CD47 could promote macrophage-mediated anti-tumor immunity in bladder cancer.

### 3.3. Enhanced Angiogenesis Is Observed in Bladder Cancer after CD47 Blockade Therapy

**Figure 3:**
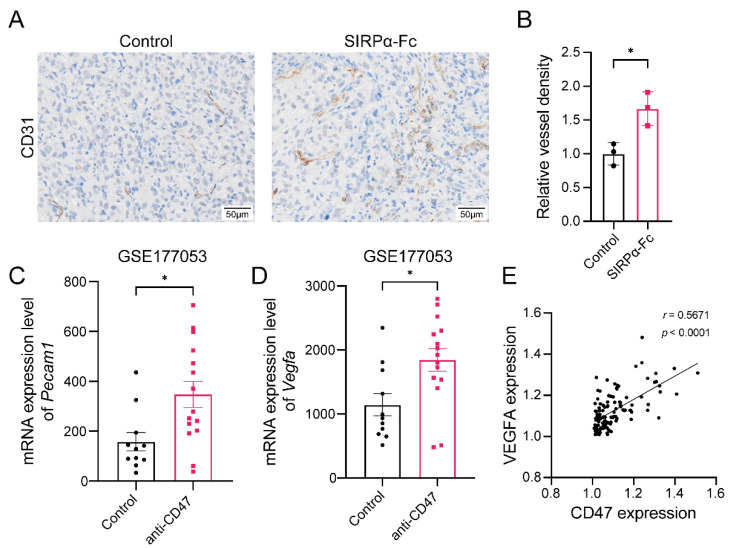
CD47 blockade therapy leads to an increase in the level of angiogenesis. (**A**) Representative images of CD31 immunohistochemistry staining of CDX tumor tissues (magnification: ×15, scale bar: 50 μm). (**B**) Relative vessel density in CDX tumor tissues after treatment with either human IgG1 isotype control or SIRPα-Fc. Data were presented as the mean ± SD and analyzed by two-tailed unpaired Student’s *t*-test. (**C**,**D**) Screening *Pecam1* and *Vegfa* mRNA expression in PBS-treated or anti-CD47-treated MC38 tumors from the GSE177053 dataset. Data were presented as the mean ± SEM and analyzed by the Mann–Whitney test. (**E**) Spearman’s correlation between expression of CD47 and VEGFA in our patient samples. *p*-values < 0.05 were considered to be statistically significant and are presented as asterisks as follows: * *p* < 0.05.

The above data demonstrate that CD47 blockade therapy leads to a decent anti-tumor effect in the bladder cancer CDX model. However, complete tumor growth control was not achieved in single-drug treatment, suggesting that there were factors existing in bladder cancer hampering the success of treatment. One of the well-known suppressive factors for tumor immunotherapy is angiogenesis, which could contribute to the formation of nonfunctional tumor blood vessels, impair anti-tumor immunity, and attenuate the efficacy of immunotherapy. Therefore, we evaluated whether angiogenesis weakens the anti-tumor effect of CD47 blockade therapy in the bladder cancer CDX model. By analyzing changes in angiogenesis through IHC staining, we found that angiogenesis was aggravated after SIRPα-Fc treatment in bladder cancer (Figure 3A,B). A similar phenomenon was found in the GSE177053 dataset studying gene expression profiling in the MC38 model treated with anti-CD47 antibodies; the expression of *Pecam1* (CD31-encoding gene) and *Vegfa* was significantly upregulated in the CD47 blockade group (*p* < 0.05) (Figure 3C,D). These results indicate that CD47 blockade therapy could lead to enhanced angiogenesis, which may hamper its anti-tumor effect. In addition, a positive correlation between the protein expression of CD47 and VEGFA was also observed in the patient tissue samples (Figure 3E), implying that there might be higher intrinsic angiogenesis levels among patients with higher CD47 expression. Thus, we reasoned that the combination of anti-angiogenic drugs with CD47 blockade might provide a superior anti-tumor effect in bladder cancer.

### 3.4. Inhibiting Angiogenesis Markedly Improves the Anti-Tumor Effect of CD47 Blockade in Bladder Cancer

**Figure 4:**
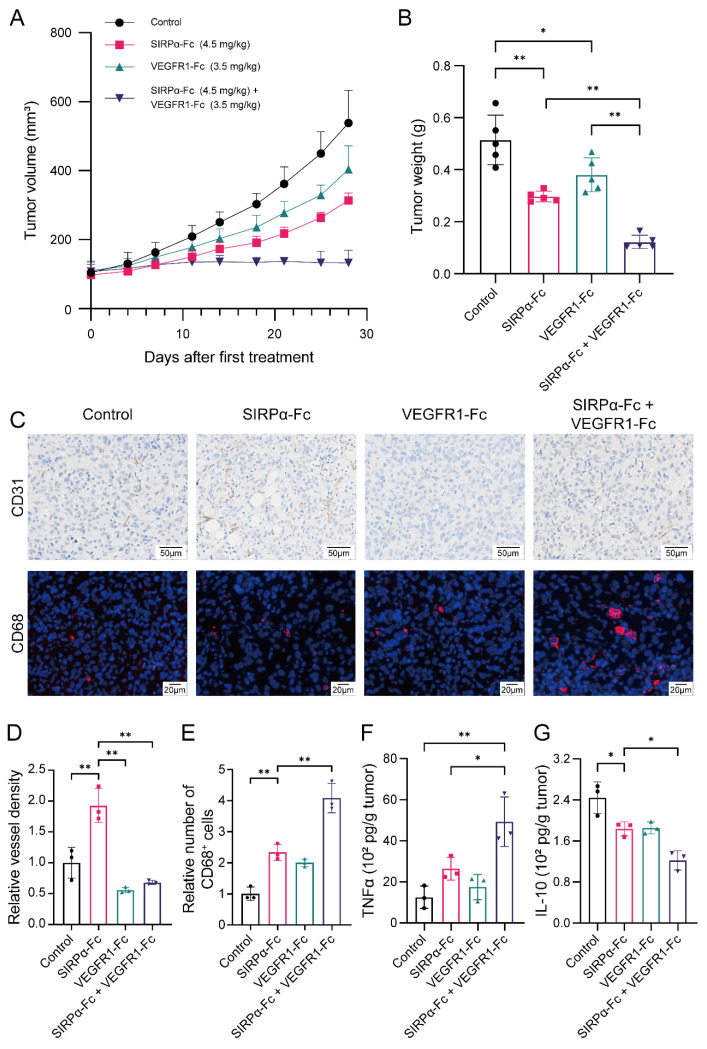
The combination of VEGFR1-Fc with SIRPα-Fc enhances the anti-tumor effect of CD47 blockade in bladder cancer CDX model. (**A**,**B**) Tumor growth in the HSPCs-CDX model after treatment with human IgG1 isotype control (2.4 mg/kg, i.p. injection twice a week), SIRPα-Fc (4.5 mg/kg, i.p. injection twice a week), VEGFR1-Fc (3.5 mg/kg, i.p. injection twice a week), or combination therapy (4.5 mg/kg SIRPα-Fc plus 3.5 mg/kg VEGFR1-Fc, i.p. injection twice a week). (**C**–**E**) Representative images of CD31 and CD68 staining of CDX tumor tissues in different treatment groups (for upper panel, magnification: ×15, scale bar: 50 μm; for lower panel, magnification: ×20, scale bar: 20 μm). Relative vessel density in CDX tumor tissues and the relative number of tumor-infiltrating macrophages in different treatment groups. (**F**,**G**) Intratumoral levels of TNF-α and IL-10 in each treatment group. Data above were presented as the mean ± SD and analyzed by ordinary one-way ANOVA. *p*-values < 0.05 were considered to be statistically significant and are presented as asterisks as follows: * *p* < 0.05 and ** *p* < 0.01.

To investigate whether combining anti-angiogenic drugs with CD47 blockade could further inhibit tumor growth, we compared the anti-tumor effect of single-agent therapy and combination therapy in the HSPCs-CDX model. Single-agent therapy with either SIRPα-Fc or VEGFR1-Fc could inhibit tumor growth to some extent but eventually, the tumor growth progressed and resisted the treatment. Conversely, combination therapy successfully restricted tumor growth and maintained anti-tumor sensitivity (Figure 4A,B). Mechanistically, combination therapy abrogated the overgrown microvessel caused by CD47 blockade and significantly promoted the infiltration of macrophages compared to single-agent therapy (*p* < 0.01) (Figure 4C–E). Combination therapy also resulted in a greater elevation of the intratumoral TNF-α level, while further decreasing the IL-10 level (Figure 4F,G). Altogether, the results suggest that inhibiting angiogenesis could markedly improve the anti-tumor effect of CD47 blockade in bladder cancer.

### 3.5. Co-Targeting CD47 and Angiogenesis Effectively Inhibits the Growth of Bladder Cancer

**Figure 5:**
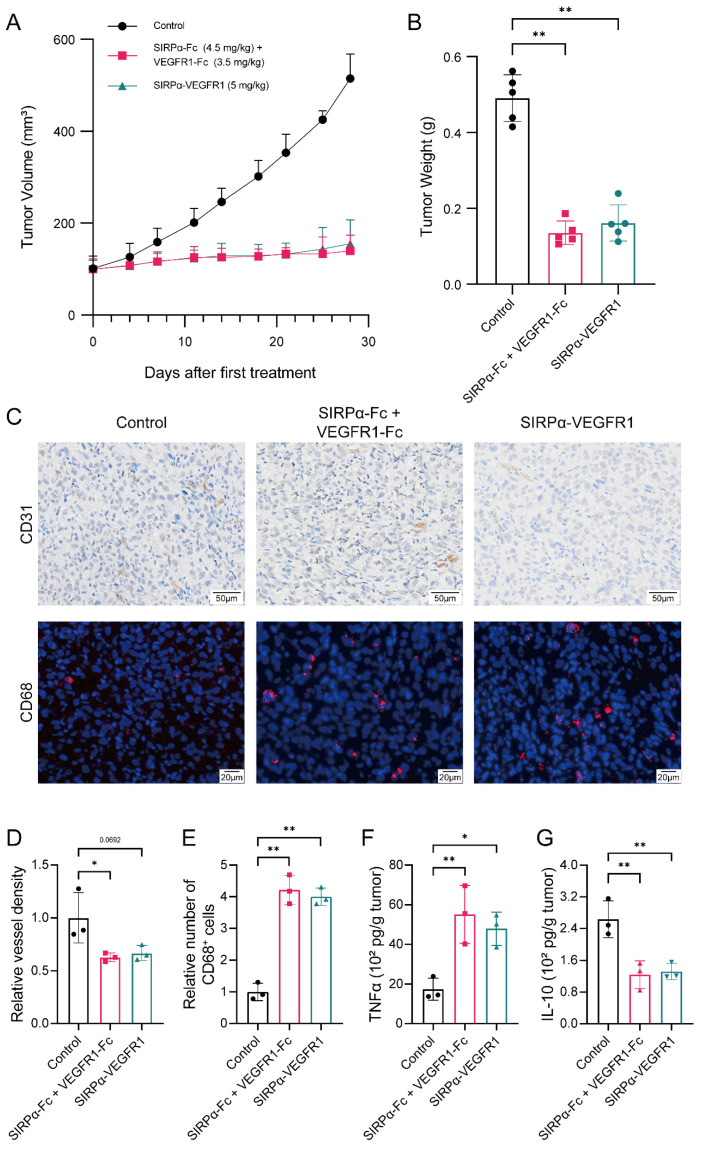
Bispecific fusion protein SIRPα-VEGFR1 effectively inhibits the growth of bladder cancer CDX model as a single agent. (**A**,**B**) Tumor size and weight of different treatment groups in the HSPCs-CDX model (2.4 mg/kg human IgG1 isotype control, i.p. injection twice a week; 4.5 mg/kg SIRPα-Fc plus 3.5 mg/kg VEGFR1-Fc, i.p. injection twice a week; and 5 mg/kg SIRPα-VEGFR1, i.p. injection twice a week). (**C**–**E**) Levels of angiogenesis and infiltrating macrophages in each treatment group (for upper panel, magnification: ×15, scale bar: 50 μm; for lower panel, magnification: ×20, scale bar: 20 μm). (**F**,**G**) Levels of TNF-α and IL-10 in CDX tumor tissues after corresponding treatment. Data above were presented as the mean ± SD and analyzed by ordinary one-way ANOVA. *p*-values < 0.05 were considered to be statistically significant and are presented as asterisks as follows: * *p* < 0.05 and ** *p* < 0.01.

After proving that the combination of CD47 blockade and anti-angiogenic drugs could elicit a potent anti-tumor effect in bladder cancer, we investigated the co-targeting effect of simultaneously blocking CD47 and angiogenesis. Co-targeting CD47 and angiogenesis was achieved by a previously developed bispecific fusion protein SIRPα-VEGFR1. In the HSPCs-CDX model, we found that SIRPα-VEGFR1 elicited an effective anti-tumor effect to a similar extent as combination therapy (Figure 5A,B). Co-targeting CD47 and angiogenesis successfully controlled tumor neovascularization and led to an immune-favorable, macrophage-infiltrating, pro-inflammatory tumor microenvironment (Figure 5C–G). In summary, dual blocking CD47 and angiogenesis effectively inhibited the growth of bladder cancer and was comparable to combination therapy.

## 4. Discussion

Clinical treatment of bladder cancer is still challenging and currently remains limited. Fortunately, the development of immunotherapy provides hope for bladder cancer patients. Bladder cancer has high levels of microsatellite instability and mutation burden, which are deemed to be preferable for immunotherapy [29,30]. Apart from BCG treatment and PD-1/PD-L1 blockades, other immunotherapy schemes such as cytokine therapy (N-803) [31], recombinant oncolytic virus therapy (CG0070) [32], and cancer vaccines [33] are also being developed and have gained decent results. N-803, for example, is an IL-15 agonist developed by ImmunityBio; its combination with BCG has been shown to be effective for patients with BCG-unresponsive bladder cancer; 71% of patients who had failed with previous therapies show a complete response with a median duration of 26.6 months [31]. However, most of the research and developments focus on the treatment of early-stage bladder cancer and those agents are administrated locally. Compared with systemic immunotherapy, they might not be suitable for patients with advanced or metastatic tumors. Therefore, it is worth investigating other immunotherapy targets for systemic immunotherapy in bladder cancer.

CD47 is an attractive immunotherapy target for counteracting tumors. The interfering CD47-SIRPα axis could be implemented by using antibodies, fusion proteins, or even small-molecule inhibitors [34]. Studies have shown that targeting CD47 with anti-CD47 or a SIRPα fusion protein containing IgG1 Fc could exert dual effects, disrupting CD47-SIRPα interaction and promoting FcγR-mediated phagocytosis, which results in an enhanced clearance of tumor cells by macrophages and other APCs [35,36]. An improved uptake of tumor antigens by APCs could also boost their antigen presentation ability and chemokine production to some extent, which could promote the activation of anti-tumor adaptive immunity [8,37]. Targeting CD47 has been widely studied in many types of cancer, including hematologic malignancies, lung cancer, colorectal cancer, and breast cancer. In bladder cancer, there have been attempts at using CD47 antibodies as a targeting domain for molecular imaging and drug delivery [38,39,40]. However, the therapeutic effect of blocking CD47 in bladder cancer remained unclear. Our study assessed the anti-tumor efficacy of CD47 blockade in bladder cancer, indicating that CD47 is a suitable immunotherapy target for bladder cancer and CD47 blockade could inhibit the growth of bladder cancer through promoting the macrophage-mediated anti-tumor immunity.

Although CD47 blockade is powerful in hematologic malignancies, its efficacy in solid tumors has been much less rewarding. Monotherapy with CD47 blockade usually could not fully inhibit the growth of solid tumors due to the “antigen sink” effect and the heterogeneity of the tumor microenvironment [41,42]. A high dose of CD47 blockade might deliver better efficacy, but it could cause RBC and platelet depletion toxicities, resulting in severe hemolysis and thrombocytopenia [10,43]. A lower dose of CD47 blockade or IgG4 Fc-based antagonist may ameliorate the adverse effects but will impair the anti-tumor efficacy [44]. To overcome these limitations, several combination strategies have been investigated. For example, several clinical studies have been initiated in past years to evaluate the tolerability and efficacy of anti-CD47 antibodies combined with azacitidine in the treatment of patients with MDS and AML [45,46]. In pre-clinical studies, it has been found that radiotherapy in combination with CD47 blockade could stimulate a potent macrophage-mediated abscopal effect in non-small cell lung cancer, lymphoma, and colon cancer models, leading to tumor control at both the irradiated and non-irradiated site [47]. Thus, combining other drugs or therapies is considered to be a potential way to supplement the loss of activity and enhance the efficacy of CD47 blockade.

**Figure 6:**
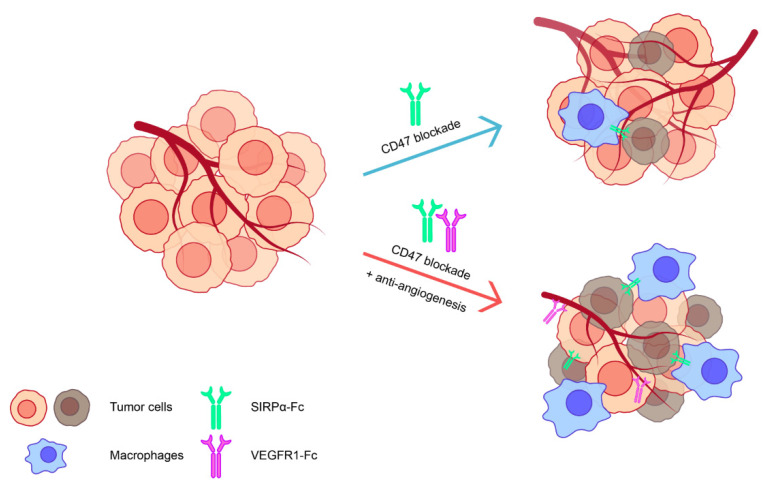
Targeting CD47 and angiogenesis exhibits a potent anti-tumor effect in bladder cancer. A graphical illustration of the synergistic effects of simultaneously targeting CD47 and angiogenesis in bladder cancer.

The immunosuppressive or “cold” tumor microenvironment is a main obstacle for the success of immunotherapy in solid tumors [18]. Tumor and immunosuppressive cells could produce suppressive cytokines and metabolites and transduce suppressive signals to inhibit the survival and activation of anti-tumor immune cells [48], while physical barriers such as an excessive accumulation of collagen and fibronectin could limit the egress of anti-tumor immune cells to tumors [49]. Multiple factors could lead to this undesirable phenomenon; our team considers that angiogenesis could be a key regulatory factor. Tumor angiogenesis is a complicated process during tumor growth. It might be an important mechanism of immunotherapy resistance, as it could hamper the delivery of drugs and cells to tumors and tune the tumor microenvironment into an immunosuppressive state [50]. Of note, angiogenesis has also been associated with CD47. Previous studies have reported that thrombospondin-1 (TSP1) could inhibit angiogenesis by interacting with CD47 in endothelial cells, whereas CD47 deficiency could promote angiogenesis [51,52]. Our results showed that CD47 blockade in bladder cancer caused enhanced angiogenesis in CDX tumor tissues, which might harm anti-tumor efficacy. Elevated angiogenesis levels could be a universal phenomenon, as we also witnessed it in some other tumor types [53,54]. We speculated that the unfavorable angiogenesis after CD47 blockade might be the result of an unintended interruption of the TSP1-CD47 pathway, but further research is needed to elucidate the underlying mechanisms. Combination therapy with CD47 blockade and anti-angiogenic drugs produces significant tumor inhibition activity in bladder cancer, which could be explained by the control or normalization of angiogenesis, as well as the further increment of tumor-infiltrating macrophages and pro-inflammatory cytokine production (Figure 6). In addition to bladder cancer, previous studies have shown that targeting CD47 and angiogenesis is also effective in inhibiting the growth of gastric cancer, glioblastoma, and non-small cell lung cancer by mediating similar mechanisms. During the treatment, we have also briefly monitored the vigor and body weight of the mice; there was no significant decline in the vigor and body weight of the mice during treatment compared to their initial state. However, more evaluations are needed to ensure the safety of combining CD47 blockade and anti-angiogenic drugs in bladder cancer. The anti-angiogenic drug we used, VEGFR1-Fc, might also have some effects on regulating anti-tumor immunity, as it traps VEGFA, which could inhibit T cells and DCs, as well as promote Treg and MDSC expansion [55]. However, these effects may not be dominant in our point of view. The dual blocking of CD47 and angiogenesis was also examined by using a bispecific fusion protein, SIRPα-VEGFR1. SIRPα-VEGFR1 could achieve similar anti-tumor effects as a combination therapy and showed that co-targeting CD47 and angiogenesis did not intervene with their individual effects. Using a bispecific fusion protein for a co-targeting strategy might have advantages in tumor targetability and efficacy. Furthermore, this could be beneficial in clinical practice, as it has lower dose mass and is more compliant as a single agent.

Our study utilized the HSPCs-CDX model to investigate the anti-tumor effects of targeting CD47 and angiogenesis in human bladder cancer. However, there exist limitations. On the one hand, although we have observed anti-tumor effects in the subcutaneous CDX model, it might not be sufficiently clinically relevant, and it remains unknown whether this combination strategy is applicable to other types of tumor models. In order to better mimic the tumor microenvironment in human patients, we could try to establish a patient-derived xenograft (PDX) model for further evaluation. Moreover, a metastatic model is also worth examining to determine if there is potential to hinder tumor colonization by targeting CD47 and angiogenesis. On the other hand, while HSPCs-derived humanized mice can mimic the human immune system to a certain extent, the development and function of various types of immune cells are incomplete, especially for T cells, B cells, and NK cells [56,57]. Therefore, we were unable to fully study the extent of the contribution of these cells to the therapeutic effect and the interaction between macrophages and these cells. Currently, there are studies being conducted to improve the deficiencies of HSPCs in humanized mice. For example, by constructing transgenic mice expressing human IL-6, IL-7, IL-15, etc., the reconstructive immune system can be more robustly developed [56,57]. More work is needed to keep improving the humanized immune system mouse model for evaluating the anti-tumor effect of immunotherapeutics in human cancer.

## 5. Conclusions

In conclusion, our works demonstrated that CD47 is a suitable target in bladder cancer, and targeting CD47 in bladder cancer could exert a decent in vitro and in vivo anti-tumor effect. However, angiogenesis is increased in CDX tumor tissues during CD47 blockade, limiting immunotherapy efficacy. Targeting CD47 and angiogenesis could further promote macrophage-mediated anti-tumor immunity and effectively inhibit the growth of CDX bladder cancer. The results showed that targeting CD47 and angiogenesis could be a potential strategy for treating bladder cancer, and anti-angiogenic drugs could improve the efficacy of anti-CD47 immunotherapy.

## Data Availability

The data are available by contacting the corresponding author upon reasonable request.

## Supplementary Materials

The following supporting information can be downloaded at: https://www.mdpi.com/article/10.3390/biomedicines12092152/s1, Table S1: Characteristics for 116 patients with bladder cancer; Table S2: Applications of antibodies used in the study; Figure S1: Gating strategy for phagocytosis assays.
