# Supplementary figures and images for: Targeting CD47 and Angiogenesis Demonstrates Effective Anti-Tumor Effect in Bladder Cancer
Source: Biomedicines. 2024 Sep 23;12(9):2152. doi: 10.3390/biomedicines12092152 (PMC11430664; doi:10.3390/biomedicines12092152)

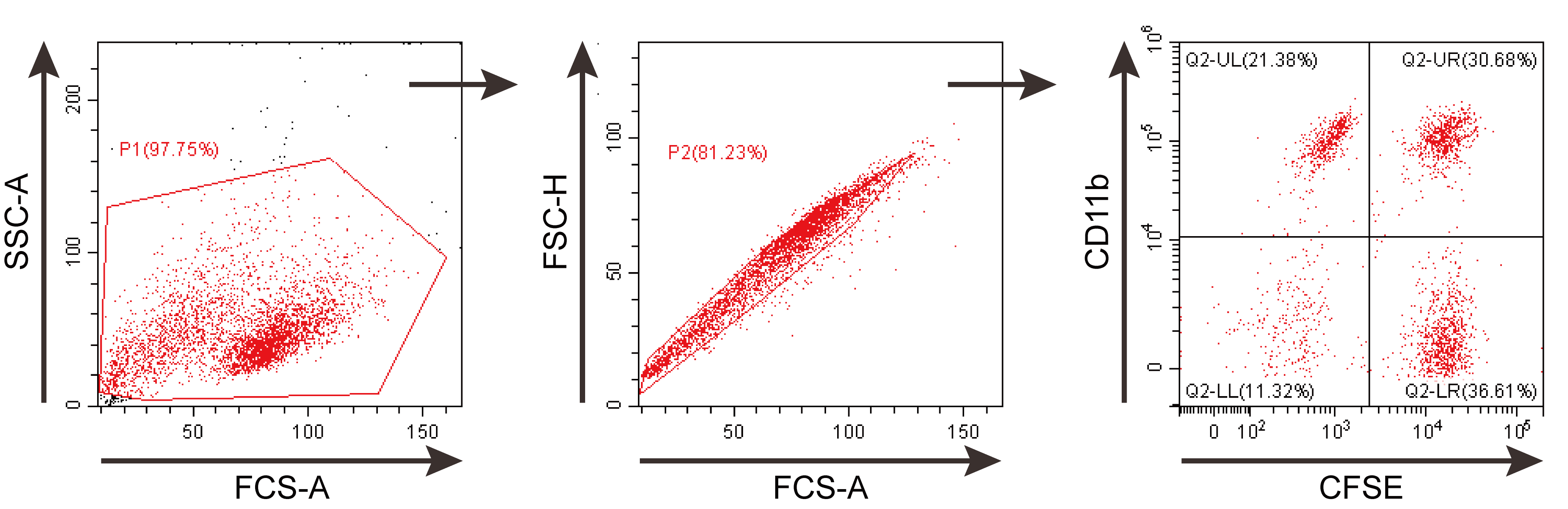

Supplement: Supplementary file 1 [file biomedicines-12-02152-s001.zip › Figure S1.png]
